# Editorial: The role of latent chronic infection in immunosenescence and inflamm-aging

**DOI:** 10.3389/fimmu.2023.1285234

**Published:** 2023-09-15

**Authors:** Andre Luis Lacerda Bachi, Asghar Abbasi, João Luiz Quaglioti Durigan, Mauro Walter Vaisberg, Rodolfo P. Vieira

**Affiliations:** ^1^ Post-Graduation Program in Health Sciences, Santo Amaro University (UNISA), São Paulo, SP, Brazil; ^2^ Division of Pulmonary & Critical Care Physiology & Medicine, The Lundquist Institute for Biomedical Innovation at Harbor-UCLA Medical Center, Torrance, CA, United States; ^3^ Laboratory of Molecular Analysis, Postgraduate Program in Rehabilitation Sciences, Faculty of Ceilândia, University of Brasilia, Brasilia, DF, Brazil; ^4^ Ear, Nose and Throat (ENT) Lab, Department of Otorhinolaryngology, Federal University of São Paulo, São Paulo, SP, Brazil; ^5^ Postgraduate Program in Human Movement and Rehabilitation and in Pharmaceutical Sciences, Evangelical University of Goias (Unievangélica), Anápolis, GO, Brazil; ^6^ Post-graduate Program in Bioengineering, Universidade Brasil, São Paulo, SP, Brazil; ^7^ Post-graduation Program in Sciences of Human Movement and Rehabilitation, Federal University of São Paulo (UNIFESP), Santos, SP, Brazil

**Keywords:** inflamm-aging, immunosenescence, infection, immunology, cytokines

The Research Topic entitled *The Role of Latent Chronic Infection in Immunosenescence and Inflamm-aging* brought interesting articles to this important topic. In fact, chronic inflammatory states play a key role in immunosenescence and in inflamm-aging. Immunosenescence is characterized by a reduced number of leukocytes as well as diminished leukocyte capacity for proliferation, activation, and pathogen defense. Inflamm-aging is characterized by structural and molecular alterations in the cells, such as receptor loss and decreased mitochondrial and intracellular organelle number, which occur as a result of aging and persistent low-grade inflammation. These alterations contribute to an increased susceptibility to age-related disorders. In this context, this Research Topic has accepted five studies. The first one was a review from Cunha et al., entitled “*Investigating population-level immunosenescence: From bench to bedside*”, in which the authors demonstrated the gold standard methods to investigate the characteristics of the immunesenescence as well as the clinical impact of such alterations on health and disease. The second study was equally a review from Rangel et al., entitled “*Human endogenous retroviruses and the inflammatory response: A vicious circle associated with health and illness*”. In this study, the authors have summarized multiple aspects of human endogenous retroviruses (HERVs’), encompassing viral and molecular aspects as well as their fusogenic properties and their impacts on health, disease, and aging. The third study accepted by this Research Topic was a mini-review from Cisneros et al., entitled “*Immune system modulation in aging: Molecular mechanisms and therapeutic targets*”. In this study, the authors reviewed the relationship between immunosenescence and inflammaging focusing on the molecular mechanisms involved in this interaction as well as possible therapeutic strategies to prevent and recover the immune response while avoiding inflammation. In the fourth study entitled “*Transfer Factor Peptides (Imuno TF^®^) modulate the lung inflammation and airway remodeling in allergic asthma*”, the authors demonstrated that a food supplement named Imuno TF^®^ unregulated the Th1 immune response while downregulated the Th2 immune response, reducing the cardinal feature of asthma (Oliveira et al.). The fifth study was entitled “*Cellular and humoral immune responses to vaccination for COVID-19 are negatively impacted by senescent T cells: a brief research report*”. In this fifth study, the authors reported not only the capacity of the CoronaVac vaccine to elicit an early cellular response followed by a humoral response but also that an increased pro-inflammatory status can drive the development of senescent T cells, which could be related to the impairment of the COVID-19 vaccination.

## Author contributions

AB: Data curation, Formal Analysis, Supervision, Writing – original draft. AA: Conceptualization, Data curation, Formal Analysis, Validation, Writing – original draft, Writing – review & editing. JD: Conceptualization, Data curation, Formal Analysis, Validation, Writing – original draft, Writing – review & editing. MV: Conceptualization, Data curation, Formal Analysis, Writing – original draft, Writing – review & editing. RV: Conceptualization, Formal Analysis, Supervision, Validation, Writing – original draft, Writing – review & editing.

